# Association of serum lipid profiles and dietary intakes of vitamin E and fiber with psoriasis severity

**DOI:** 10.22088/cjim.12.4.606

**Published:** 2021

**Authors:** Mohammad Javad Yazdanpanah, Sadegh Vahabi-Amlashi, Mohsen Nematy, Neda Shaelaei, Seyed Amir Reza Mohajeri, Zahra Tafazzoli

**Affiliations:** 1 Cutaneous Leishmaniasis Research Center, Mashhad University of Medical Sciences, Mashhad, Iran; 2 Department of Nutrition and Biochemistry, Faculty of Medicine, Mashhad University of Medical Sciences, Mashhad, Iran; 3 Faculty of Medicine, Mashhad University of Medical Sciences, Mashhad, Iran

**Keywords:** Psoriasis, FFQ, Lipid profile, Oxidative stress, BMI, PASI score

## Abstract

**Background:**

Dyslipidemia has been reportedly associated with an increased risk of atherosclerosis among psoriatic patients. Dietary intake can be a key factor in the pathophysiology of psoriasis. Herein, we assessed serum lipid profile and dietary intake in psoriatic patients, in comparison with healthy subjects.

**Methods:**

In this case-control study, 45 psoriatic patients and 43 healthy controls were evaluated. We estimated the macro/micronutrient intakes and energy, using a food frequency questionnaire (FFQ). Anthropometric parameters and serum levels of triglyceride (TG), high-density lipoproteins (HDL), low-density lipoproteins (LDL), and very low-density lipoproteins were assessed. The case group was categorized by severity measured by PASI score (mild<10, moderate 10-20, severe >20). Diet plan 6.0 was used to analyze FFQs and data were analyzed in SPSS 16.0, with p<0.05 considered significant.

**Results:**

The case group had markedly higher body mass index (BMI), LDL, and cholesterol and significantly lower HDL compared with controls (p<0.05). Carbohydrate, energy, fat intakes were significantly higher in cases, while folate, fiber, and vitamin E intakes were significantly lower in the case group, compared with the control group (p<0.05). BMI, cholesterol, and triglyceride values and dietary intakes of fiber and vitamin E were significantly associated with severity of psoriasis (p<0.05).

**Conclusion:**

Serum lipid profile and dietary intake are substantially important in psoriasis severity. Therefore, close monitoring of lipid profile and BMI during admission and follow-up and dietary modification can improve the severity of psoriasis.

Psoriasis is a relatively common chronic inflammatory disease of the skin, characterized by plaques surfaced with scales on both upper and lower limbs extensors, scalp, nails, umbilicus, lumbosacral and palmo-plantar areas (1, 2). It affects 1.5-3.0% of the general population worldwide (3). Although psoriasis appears to be a hereditary disease, several environmental risk factors, especially unhealthy dietary habits, have been reported to be involved in its pathophysiology (4). Inflammatory reactions mediated by T lymphocytes (CD4 and CD8) increase the levels of cytokines such as interferon-gamma (INF-γ), interleukins (IL) 1, 2, 6, and 17, and tumor necrosis factor-alpha (TNF-α). This in turn leads to hyperproliferation of macrophages and keratinocytes and increased skin lesions in psoriatic patients (1, 5). These proinflammatory cytokines are involved in the pathophysiology of a range of conditions including cardiovascular diseases, obesity, hypertension, diabetes mellitus, and inflammatory bowel disease (6-9).

The inflammatory responses that underlie atherosclerotic lesions and psoriasis seem to have a substantial overlap. It has been proposed that the increased atherosclerosis risk among psoriatic patients may be due to abnormal lipid metabolism (10). However, it remains controversial whether abnormal lipid metabolism or psoriasis leads to this inflammatory state (11, 12). A large body of evidence reports the crucial role of unhealthy dietary patterns in the pro-inflammatory state (13). However, there is a paucity of evidence regarding the interrelationship of nutritional status, abnormal lipid metabolism and psoriasis (14).

Herein, we aimed to investigate the serum lipid profile and micro/macronutrient dietary intakes in patients with psoriasis who had not received any medications that could alter serum lipids, in comparison with healthy individuals. We hypothesized that abnormal serum lipid profile and dietary intakes of some micro/macronutrient may make a major contribution to the severity of psoriasis.

## Methods

**Study Settings and Population: **This case-control study was conducted among 45 patients with psoriasis (case group) and 45 sex- and age-matched individuals that were otherwise healthy (control group) who referred to the outpatient dermatology clinics of Qaem and Imam Reza Hospitals, Mashhad, Iran, between June 2015 and December 2017.

Keeping an alpha=0.05 and a beta=0.2 with a study power of 80%, the sample size was calculated to be 84 subjects (42 in each group), with respect to the mean LDL levels reported in a previous study (15). However, we extended the sample size to 45 subjects in each group, considering a few cases might be lost to follow.

The diagnosis of psoriasis was made clinically and suspicious cases were histologically confirmed. Subjects who received any treatments that could alter serum lipid profile such as anti-lipids, systemic corticosteroids, cyclosporine and, beta-blockers were excluded from the study. Patients with other skin disorders, malignancy, systemic or cardiovascular diseases such as familial hyperlipidemia, hypothyroidism, chronic kidney disease, hypertension, diabetes mellitus, and hyperlipidemia were excluded from the study. The pregnant or breastfeeding women were also excluded.

This study was approved by the ethics committee at the university and was done according to the codes of ethical conduct expressed in the Declaration of Helsinki. All participants signed informed written consent prior to their enrollment.

**Clinical and Laboratory Assessments: **All eligible individuals were assessed through complete medical history and thorough physical examination by a single dermatologist. Demographic, anthropometric, and clinical data were measured in standard settings and recorded in checklists for all subjects. Body mass index (BMI) was calculated using the measured values of weight and height.

We measured the clinical severity of psoriasis based on psoriasis area and severity index (PASI). To investigate the possible associations between serum lipids, nutritional intake, and psoriasis severity, we categorized patients based on their disease severity into three subgroups including mild (PASI<10), moderate (PASI of 10-20), and severe (PASI>20).

After 12 hours of fasting, we gathered blood samples in Vacutainer® tubes and centrifuged them at 5,000 g for 15 m at 4° Celsius. For further analysis, we froze the aliquots of serum at ˗80° Celsius after separating for further analysis. A full fasted profile of serum lipids including total cholesterol, triglycerides, low-density lipoprotein cholesterol (LDL-C), very low-density lipoprotein (VLDL), and high-density lipoprotein cholesterol (HDL-C), as well as fasting plasma glucose (FPG) were measured in all subjects. Serum lipids and FPG titers were enzymatically assessed with commercial kits by BT-3000 auto-analyzer (Biotechnica, Rome, Italy).

**Dietary Assessment: **Dietary assessment was performed by a certified dietitian through face-to-face interviews by the validated Persian version of semi-quantitative 160-item food frequency questionnaire (FFQ) that has been modified according to Iranian food items (16). A single dietitian asked all subjects to report each food and drink items with a portion size consumed during the past year per day, per week or per month. Then, portion sizes were converted to SI units. Nutrient compositions of the consumed foods were analyzed using Diet Plan 6.0 software (Forest field software Ltd, Horsham, UK).

**Statistical Analysis: **Data were analyzed in SPSS (Version 16 for Windows, IBM Statistics, Chicago, IL). Quantitative parameters were presented as means ± standard deviation (SD). Group comparisons were carried out by independent samples t-test or one-way ANOVA for normally distributed data. For comparison of non-normally distributed data, Mann-Whitney and Kruskal-Wallis tests were used. Chi-square test was used for categorical parameters. A p<0.05 was considered significant.

## Results

**Demographic and Biochemical Characteristics: **Overall, 45 patients in the case group and 43 control subjects completed the study and two control subjects were excluded due to missing data. The case group had an average age of 40.4±9.2 years, while the average age in the control group was 38.7±9.9 years (P=0.41). Sixteen (35.6%) subjects in the case group and 21(48.8%) in the controls were males (P=0.21). Table-1 compares the demographic characteristics and biochemical factors between cases and controls. Serum cholesterol and LDL levels were significantly higher among the case group, in comparison with the control group (P<0.05). 

Serum HDL was significantly lower in the case group compared with the control group (41.8±8.8 vs. 47.3±12.2 mg/dl, P=0.02). In addition, patients with psoriasis showed higher BMI values compared with the control group (26.9±2.7 versus 24.4±4.8 kg/m^2^, P=0.01).

**Dietary Intake: **As table-2 shows, patients with psoriasis showed higher intakes of carbohydrates, fats, fiber, energy, vitamin E and folate compared with controls (p<0.05). The dietary intakes of protein, vitamin A, and potassium had no significant difference between two study groups.

**Severity of Psoriasis: **Comparison of anthropometric, biochemical and dietary intake parameters between patients with different severity of psoriasis is detailed in table-3. As the table implies, there were significant associations between the severity of psoriasis and BMI, cholesterol, and triglyceride (p<0.05), indicating an increase in these parameters with the increase in severity of psoriasis. Increased dietary intake of fiber and vitamin E was also significantly associated with lower disease severity (p<0.05). 

There was a gradual decline in dietary fiber intake with the increase in severity of psoriasis (P=0.44). When comparing the patients with severe psoriasis to those with mild psoriasis, we observed a significant difference in BMI, cholesterol, and triglycerides values (p<0.05). 

Besides, in patients with mild psoriasis, the dietary intake of fiber and vitamin E was significantly lower compared to those with severe psoriasis (P<0.05).

**Table 1:** Demographic and biochemical characteristics in the two groups

Variable	Case (n=45)	Control (n=43)	P-value
**Age (y)**	40.4 ± 9.2	38.7 ± 9.9	0.41
**Gender** **(male)**	16 (35.6%)	21 (48.8%)	0.21*
**BMI (kg/m** ^2^ **)**	26.9±2.7	24.4 ± 4.8	0.01
**Total cholesterol (mg/dl)**	193.2±30.1	175.9±40.2	0.03
**Serum triglycerides (mg/dl)**	117.3±49.5	104.5±50.2	0.24
**LDL-C (mg/dl)**	129.2±27.0	112.2±28.7	0.006
**HDL-C (mg/dl)**	41.8±8.8	47.3±12.2	0.02
**VLDL (mg/dl)**	22.3±20.3	16.5±17.2	0.24
**FPG (mg/dl)**	86.6±10.5	83.8±7.9	0.02

LDL-c: Low-density lipoprotein cholesterol, HDL-C: High-density lipoprotein cholesterol, VLDL: Very low-density lipoprotein cholesterol, FPG: Fasting plasma glucose; *Chi-square test was used to compare the gender distribution between case and control groups, student sample t-test and Mann-Whitney tests were used to compare qualitative and quantitative (normal and non-normal) variables, respectively.

**Table 2:** Comparison of dietary intakes between the case and the control groups

Variable	Case (n=45)	Control (n=43)	P-value
**Protein (g)**	79.1 ± 20.4	73.5 ± 23.8	0.30
**Carbohydrates(g)**	289.8 ± 94.4	245.1 ± 72.4	0.04*
**Fats (g)**	105.4 ± 26.1	82.6 ± 25.7	<0.001*
**Fiber (g)**	11.6 ±7.9	14.7±7.2	0.03*
**Energy (Kcal)**	2424.7±654.6	2017.9±575.2	0.01*
**Vitamin A (µg)**	1038.5±626.8	724.2±353.2	0.11
**Vitamin E (mg)**	5.9±3.8	10.3 ± 9.1	0.049*
**Folate (µg)**	254.5±160.1	350.3±178.0	0.01*
**Potassium (mg)**	3239.6±1587.3	2676.8±1012.2	0.07

* Mann-Whitney tests were used to compare variables between case and control groups.

**Table 3:** Comparison of anthropometric, biochemical, and nutritional data between patients with different severities of psoriasis

**Parameter**		**Mild (PASI<10) (n=27)**		**Moderate (PASI:10-20) (n=8)**		**Severe (PASI>20) (n=10)**		P*		P**
Age (y)		39.4 ± 11.2		39.9 ± 10.2		43.9 ± 6.4		0.60		
Male sex		7 (25.9%)		3 (37.5%)		6 (60.0%)		0.16^§^		
BMI (kg/m2)		26.2 ± 3.0		26.9 ± 2.0		28.7 ± 1.2		0.04		0.02
TC (mg/dl)		184.4 ± 34.8		205.5 ± 9.4		207.4 ± 16.7		0.049		0.01
TG (mg/dl)		96.0 ± 39.4		122.0 ± 43.0		170.9 ± 38.5		<0.001		<0.001
LDL-C (mg/dl)		122.6 ± 28.5		146.5 ± 25.6		133.0 ± 17.3		0.08		0.29
HDL-C (mg/dl)		42.0 ± 10.6		44.6 ± 4.7		39.0 ± 4.7		0.41		0.40
VLDL (mg/dl)		19.8 ± 11.5		14.4 ± 24.0		35.4 ± 30.5		0.05		0.14
FPG (mg/dl)		88.7 ± 11.1		83.4 ± 11.5		83.7 ± 7.1		0.28		0.12
**Nutrients**										
Protein (g)		78.1 ± 24.5		81.3 ± 17.4		80.1 ± 7.0		0.92		0.81
Carbohydrates(g)		287.3 ± 100.6		273.6 ± 99.9		309.7 ± 76.8		0.71		0.53
Fats (g)		103.5 ± 30.8		104.5 ± 24.9		111.4 ± 8.2		0.54		0.26
Fiber (g)		13.9 ± 9.3		9.6 ± 4.4		7.0 ± 1.1		0.002		0.001
Energy (Kcal)		2393.1 ± 749.4		2360.0 ± 642.7		2561.8 ± 351.5		0.76		0.50
Vitamin A (µg)		1132.1 ± 676.8		827.4 ± 460.9		954.7 ± 598.7		0.58		0.91
Vitamin E (mg)		7.1 ± 4.4		4.9 ± 1.0		3.6 ± 1.7		0.04		0.02
Folate (µg)		282.7 ± 198.8		227.5 ± 62.0		200.1 ± 42.1		0.44		0.22
Potassium (mg)		3223.2 ± 1827.8		2739.0 ± 1062.2		3684.4 ± 1160.3		0.46		0.37

* Comparison among psoriasis patients with mild, moderate and severe based on the PASI score using one-way ANOVA

** This p-value represents the comparison of demographic and biochemical parameters, as well as nutritional intake between the patients with mild and severe psoriasis

TC: Total cholesterol, TG: Triglycerides, LDL-C: Low-density lipoprotein cholesterol, HDL-C: High-density lipoprotein cholesterol, VLDL: Very low-density lipoprotein cholesterol, FPG: Fasting plasma glucose

§ Chi-square test was used to compare gender distribution between patients with mild and severe psoriasis.

## Discussion

We seek to assess the relationship of dietary macro/micronutrient intake and lipid profile with psoriasis. In addition, we investigated the relationship of anthropometric data, lipid profile, and dietary intake between patients with different severities of psoriasis.

Our results indicated that BMI and serum levels of cholesterol, HDL and LDL significantly differed between the two groups. Moreover, the case group showed a higher intake of carbohydrates, fats, fiber, energy, vitamin E, and folate compared with those of the controls. We observed a significant and gradual growth with increase in disease severity in many factors such as BMI and serum cholesterol and triglyceride, while a significant negative growth with increase in disease severity was observed in dietary intake of vitamin E and fiber. This is in line with previous reports indicating the association between psoriasis and dietary intakes of vitamin E and fiber (17, 18). Consistent with our findings, Barrea et al. (18) observed that patients with psoriasis had higher serum LDL, triglyceride and liver enzymes compared with healthy individuals. In addition, they found a marked relation between serum triglycerides and psoriasis severity measured by PASI score. The possible link between psoriasis and liver disorders such as non-alcoholic fatty liver disease was argued in previous studies and it might be due to the hyperinflammatory state of both psoriasis and steatohepatitis (19).

It is also suggested that macronutrients such as simple carbohydrates and saturated fats may play a role in proinflammatory states; whereas fiber and vitamin E are in relationship with decreased inflammation levels (13). In the present study, the association between the severity of psoriasis measured by PASI score and dietary fiber and vitamin E intakes was mainly preserved. It is stated that both the general diet and single food components may make major contributions to the etiology and pathogenesis of psoriasis (20). Most of the previous studies investigated the association of psoriasis and dietary patterns or dietary micronutrient intakes (21). However, since some of the dietary patterns comprised similar and correlated nutrients, it is very challenging to point out a sole micronutrient or a dietary pattern as the responsible factor (22). In addition, in some eastern countries like ours, it is believed that traditional herbs can improve psoriasis symptoms, and, in some instances, patients might not declare and report using these herbs, which can affect the study results.

In contrast to the study on dietary intake in patients with psoriasis using the 2003–2006 National Health and Nutrition Examination Survey (NHANES) data (21), we observed that psoriatic patients consumed more carbohydrates than healthy individuals did. Higher intake of sugar may lead to an inflammatory state in psoriasis through elevated inflammatory cytokines and pro-inflammatory adipokine profile (23). In addition, some reports have indicated a link between psoriasis, insulin resistance, and type 2 diabetes (24). Sex, age, and education have been established to have crucial effects on food selection behaviors (25). Dietary profiles of females showed high levels of carbohydrate intake, including vegetable and fruits (26). Psoriasis has been reportedly associated with lower intake of simple carbohydrates according to 2003-2006 NHANES, which is in contrast to some previous studies (18). Likewise, in our study, we observed that psoriatic patients consumed more carbohydrates than the controls. We also observed that psoriatic patients were more obese than healthy controls. Likewise, Yamashita et al. found that BMI was significantly different between 70 Japanese patients with psoriasis vulgaris and 70 control subjects (27). In addition, it is worth noting that the levels of LDL were significantly higher among patients with psoriasis compared with the controls in our study, which is in line with the findings of Farshchian et al. (15).

In this study, we observed a marked association between dietary intake and psoriasis. However, this association might be affected by several confounders including age, sex, cardiometabolic risk factors, the level of education, and physical activity status (27).

This work had several limitations. For instance, our sample size could be regarded as a relatively small one; conducting a study with a larger sample size seems feasible. Secondly, we used a validated 160-item semi-quantitative FFQ for recording the patient's dietary intake. Although this questionnaire is modified for Iranian foods and drinks, there are more accurate questionnaires to use for patients' dietary intakes (28). Moreover, although the groups were matched for sex and age, we could not match them in terms of BMI and this could have affected the results. Finally, some of the patients might have clandestinely used herbal and palliative treatments, which could potentially reduce disease severity and might lead to concealment of the association between disease severity and dietary intake. However, we asked for any probable tradition or palliative treatments from the patients. 

Our sample of Iranian psoriatic patients showed higher serum cholesterol and LDL levels and lower levels of HDL in comparison with healthy control subjects. Moreover, psoriatic patients had higher intakes of carbohydrates, fats, fiber, energy, vitamin E and folate compared with those of the controls. In the patients with psoriasis, higher BMI, cholesterol, and triglycerides were associated with higher clinical severity that might be due to abnormal lipid metabolism.

Low consumption of fiber and vitamin E can be stated as a major predictor of clinical severity in psoriatic subjects, through its role in inflammatory processes. Implications of these results in both the pathogenesis and treatment of psoriatic symptoms should be identified in future studies. Future multi-centered and large-scaled studies with dietary intervention studies are required to prove the role of macro/micronutrient intake in treatment/pathophysiology of psoriasis.
